# Are there differences in birth weight according to sex and associations with maternal exposure to air pollutants? A cohort study: Existem diferenças no peso ao nascer de acordo com sexo e associações com exposição materna a poluentes do ar? Estudo de coorte

**DOI:** 10.1590/1516-3180.2016.0262100317

**Published:** 2017-07-31

**Authors:** Luiz Fernando Costa Nascimento, Adrian Blanco Machin, Djalma Antonio Almeida dos Santos

**Affiliations:** I PhD. Researcher, Department of Energy, Universidade Estadual Paulista (UNESP), Guaratinguetá (SP), and Assistant Professor, Department of Medicine, Universidade de Taubaté (UNITAU), Taubaté (SP), Brazil.; II BSc. Postgraduate Student, Department of Energy, Universidade Estadual Paulista (UNESP), Guaratinguetá (SP), Brazil.; III MD. Postgraduate Student, Department of Energy, Universidade Estadual Paulista (UNESP), Guaratinguetá (SP), Brazil.

**Keywords:** Air pollutants, Ozone, Infant, low birth weight, Particulate matter, Air pollution

## Abstract

**CONTEXT AND OBJECTIVE:**

Several effects of exposure to air pollutants on human health are known. The aim of this study was to identify whether exposure of pregnant women to air pollutants contributes towards low birth weight and which sex is more affected.

**DESIGN AND SETTING:**

Longitudinal study using data on newborns from mothers living in São José do Rio Preto (SP) who were exposed to air pollutants in 2012-2013.

**METHODS:**

A hierarchical model on three levels was built using maternal and newborn variables and environmental concentrations of particulate matter, ozone and nitrogen dioxide in quartiles. Preterm newborns, twins and newborns with birth defects were excluded and exposure windows of 30, 60 and 90 days before delivery were considered.

**RESULTS:**

8,948 newborns were included: 4,491 males (50.2%) and 4,457 females (49.8%); 301 newborns presented low birth weight (3.4%). The mean weight differed between males (3281.0 g) and females (3146.4 g) (P < 0.001). Exposure to ozone was significantly associated with low birth weight in both sexes in the 30-day window (odds ratio, OR = 1.38) and 90-day window (OR = 1.48); and among females, in the 30-day window (OR = 1.58) and 90-day window (OR = 1.59). Exposure to particulate matter had a paradoxical protective effect. No association was found among male newborns.

**CONCLUSIONS:**

Female newborns showed greater susceptibility to maternal exposure to air pollutants. Studies on low birth weight in relation to maternal exposure to air pollutants should deal with males and females separately.

## RESUMO

**CONTEXTO E OBJETIVO:**

São vários os efeitos da exposição a poluentes do ar na saúde humana. O objetivo deste estudo foi identificar se a exposição da gestante contribui para o baixo peso ao nascer e qual o sexo mais acometido.

**TIPO DE ESTUDO:**

Estudo longitudinal com dados de recém-nascidos de mães residentes em São José do Rio Preto (SP) com exposição a poluentes do ar em 2012 e 2013.

**MÉTODOS:**

Foi construído modelo hierarquizado em três níveis com variáveis maternas, do recém-nascido e concentrações de material particulado, ozônio e dióxido de nitrogênio, em quartis. Foram excluídos recém-nascidos prematuros, gemelares ou com malformações e consideradas janelas de exposição de 30, 60 e 90 dias anteriores ao parto.

**RESULTADOS:**

Foram incluídos 8.948 recém-nascidos, 4.491 do sexo masculino (50,2%) e 4.457 do feminino (49,8%), e identificados 301 recém-nascidos com baixo peso (3,4%). Os pesos médios foram diferentes entre o sexo masculino (3.281,0 g) e o feminino (3.146,4 g) (P < 0,001). Exposição ao ozônio esteve associada significativamente ao baixo peso ao nascer em ambos os sexos nas janelas de 30 dias (*odds ratio*, OR = 1,38) e 90 dias (OR = 1,48) e, no sexo feminino, nas janelas de 30 dias (OR = 1,58) e 90 dias (OR =1,59). Exposição ao material particulado teve efeito protetor paradoxal. Não houve associação no sexo masculino.

**CONCLUSÕES:**

Houve maior susceptibilidade do sexo feminino aos poluentes a partir da exposição materna. Estudos sobre baixo peso ao nascer segundo exposição materna a poluentes do ar devem separar sexo masculino e feminino.

## INTRODUCTION

Low birth weight (LBW), defined as birth weight less than 2,500 g and as small for gestational age, is a manifestation of intrauterine growth restriction, and it is a predictor of morbidity and mortality in the first year of life. LBW may be caused by changes to placental blood flow, weight gain deficit during pregnancy and active and passive smoking involving pregnant women.^1^ In addition to these factors, the mother’s exposure to air pollutants has also been identified as associated with LBW. The pollutants that have been associated with this outcome include particulate matter,^2^^,^^3^^,^^4^ sulfur dioxide,^5^^,^^6^^,^^7^ carbon monoxide,^3^^,^^4^^,^^8^ nitrogen dioxide^3^ and ozone.^4^^,^^6^

Fetuses, in particular, are considered highly susceptible to a variety of pollutants because of their physiological immaturity. Moreover, patterns of exposure occurring in certain windows that are sensitive periods for development because of higher rates of both cell proliferations and metabolic changes may increase fetal susceptibility.^9^

Regarding these windows, it seems that the effect of exposure to air pollutants may be greatest during the last trimester of pregnancy. This effect would be similar to that of active or passive maternal smoking, i.e. it would influence birth weight. The explanation for this is that fetal weight gain is very sharp from the 28^th^ week of pregnancy onwards and involves release of hormones such as corticotrophin-releasing hormone (CRH) and placental adrenocorticotropic hormone (ACTH).^10^^,^^11^

Gender differences have been identified, such as higher birth weight among males, greater lung maturity among females and increased risk of neonatal and child mortality and increased risk of preterm birth among males.^12^ Similarly, studies have been developed in an attempt to identify differences in the responses of males and females regarding the association between maternal exposure to air pollutants and low birth weight. Some studies has shown that males are more affected, while others have shown that females are more affected and yet others have not shown any association with the sex of the newborn.^12^^,^^13^^,^^14^^,^^15^

## OBJECTIVE

The objective of this study was to identify possible differences in birth weight, according to sex, associated with maternal exposure to air pollutants in São José do Rio Preto (SP).

## METHODS

This was a longitudinal study on live birth data carried out in São José do Rio Preto, covering the period from January 1, 2012, to December 31, 2013. The data were obtained from the Brazilian Live Births Information System (Sistema de Informações sobre Nascidos Vivos, SINASC).^16^ Newborns from pregnancies that lasted for 37 weeks or more were selected for this data set. Twin deliveries and those with any type of congenital anomalies were excluded.

São José do Rio Preto is located in the state of São Paulo at 20° 49’ S and 49° 22’ W, at a distance of about 440 km from the state capital and the average annual temperature is 23.6°. The population comprises around 430,000 inhabitants in a 430-km^2^ area. The municipality had an automobile fleet of just over 320,000 vehicles in 2012, and an urbanization rate of around 94.1%. It has 157 healthcare facilities. Its Human Development Index (HDI) is 0.79 and it is an important production center for sugar and alcohol.^17^

The mother and child variables included in this study were as follows: the mother’s age (in years) was categorized as up to 19 years of age and above 34 years (at risk) or 20-34 years (non-risk); the number of children alive was categorized as none (non-risk) or one or more (at risk); the mother’s marital status was categorized as living with partner or husband (non-risk) or living alone (at risk); the mother’s education level was categorized as elementary education, i.e. up to 8 years of schooling (at risk), or beyond elementary level, i.e. 9 years of schooling or more (non-risk); the prenatal number of consultations was categorized as 0 to 6 (at risk) or 7 or more (non-risk); the newborn’s sex (male or female); the newborn’s weight (in grams) was categorized as LBW or normal weight (i.e. weighing greater than or equal to 2,500 g); and the type of delivery (vaginal or cesarean section).

The environmental variables comprised the average concentrations of particulate matter less than 10 μ of aerodynamic diameter (PM10); and nitrogen dioxide (NO_2_) and ozone (O_3_), quantified in μg/m^3^. These were measured by the São Paulo state environmental agency (CETESB), at its location in the municipality. These concentrations were then transformed into mean exposures over 30, 60 and 90-day windows prior to the newborns’ delivery.

Hierarchical unconditional logistic regression at three levels (distal, intermediate and proximal) was built to quantify the effect of pregnant women’s exposure to air pollutants on the weight of their newborns, represented by the chance of having a newborn with low weight according to certain conditions.

The variables at the distal level (maternal age, parity, marital status and educational level) were analyzed in univariate analysis together with the birth weight, which was categorized as normal or LBW. If P < 0.10, the variables were kept for multivariable analysis and were then maintained at this level if P < 0.05.

Following this, we analyzed the variable at the intermediate level (prenatal number of consultations). The next step was to analyze this variable using the variables from the previous level that presented P < 0.05. The variable of prenatal number of consultations would be adjusted in accordance with the variables of the previous level. The hierarchical model was built with two levels, comprising the distal and intermediate variables.

Next, we analyzed the proximal variables, which consisted of the pollutant concentrations categorized as non-risk. If the concentration was in first quartile, it was considered to be a reference, while the other quartiles made up the at-risk group. The distal variables with P < 0.05 were kept at this level, as described above.

The analyses were performed considering both sexes and then only with males and, separately, only with females. These analyses were performed with pollutants categorized into quartiles and adjusted in accordance with the distal and intermediate level variables.

The statistical analysis was performed using the Statistical Package for the Social Sciences (SPSS), version 17. The significance level used was alpha = 5%.

This study was conducted using data available in a public access database without the possibility of identifying the subject. Thus, it was not submitted to a research ethics committee for approval.

## RESULTS

The study included 8,948 infants, consisting of 4,491 males (50.2%) and 4,457 females (49.8%), born between January 1^st^, 2012, and December 31^st^, 2013. The mean birth weight was 3213.9 g (standard deviation, SD = 416.4), with a range from 870 g to 5420 g. There were 301 newborns with low birth weight (3.4%), according to the exclusion criteria, such as pregnancy to term, single pregnancy and no malformation. The average weights according to the newborn’s sex were 3281.0 g (standard deviation, SD = 419.6) for males and 3146.4 g (SD = 402.0) for females (P < 0.001).

The odds ratio (ORs) and respective 95% confidence intervals (95% CIs) of the independent variables according to low birth weight and normal weight among the newborns are shown in Table 1. It was found that there was a significantly higher chance of low birth weight among female newborns (OR = 1.39) and among the newborns of mothers without a partner (OR = 1.28), mothers who made fewer prenatal care visits (OR = 2.45), mothers whose educational level was lower (OR = 1.59) and mothers who were under the age of 20 years and over the age of 34 years (OR = 1.29).

**Table 1.**
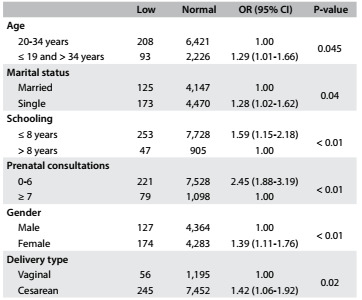
Odds ratios (OR) with 95% confidence intervals (95% CI) and P-values of the maternal variables of age, education, marital status, number of prenatal consultations and delivery type and the newborns’ sex, according to birth weight (low or normal). São José do Rio Preto, Brazil, 2012-2013

The distribution of mean pollutant levels according to 30, 60 and 90-day windows before the newborns’ delivery is shown in Table 2. The maximum values for these concentrations did not exceed the values that are considered acceptable according to state of São Paulo Decree-Law no. 59,113, of April 23, 2013.^18^

**Table 2.**
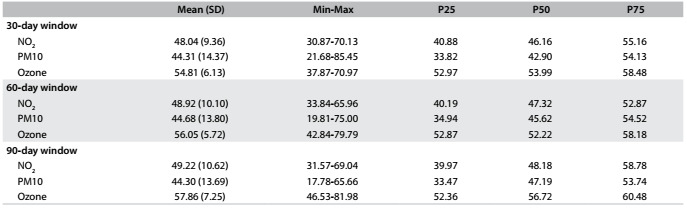
Mean concentrations of the pollutants NO_2_. PM10 and ozone (in μg/m^3^) with standard deviations (SD), minimum values (Min), maximum values (Max) and percentiles 25 (P25), 50 (P50) and 75 (P75), during the 30, 60 and 90-day windows prior to the newborns’ delivery. São José do Rio Preto, Brazil, 2012-2013

Maternal education level, maternal age and marital status were variables from the distal level with P ≤ 0.10. Maternal education level remained with P < 0.05 in the multivariate analysis, but marital status showed P = 0.07. However, because this was close to the significance level, this variable was retained due to its importance.

Inclusion of the intermediate-level variable (number of consultations), to be adjusted in accordance with the variables of the previous level, did not change its statistical significance. Thus, the hierarchical model contained two levels, comprising the variables of marital status, maternal education level and number of consultations.

Following this, the variables of the proximal level were introduced, i.e. pollutant concentrations in the 30, 60 and 90-day windows prior to the newborns’ delivery, in two classes: at-risk and non-risk. The pollutants were included in the analysis one by one according to the 30, 60 and 90-day windows. Multi-pollutant analyses were carried out on the pollutants that showed P < 0.10 in the previous step and these variables were retained if P < 0.05, after adjustment for the possible variables of the same proximal level and those of the distal and intermediate levels.

The ORs according to exposure to pollutants, for both sexes, are shown in Table 3. Exposure to ozone was associated with low birth weight in the 30 and 90-day windows before the newborns’ delivery and exposure to particulate matter was shown, paradoxically, to be protective against exposure to low birth weight in the 90-day window prior to the newborns’ delivery. Ozone exposure was associated with the 30-day window (OR = 1.39; 95% CI: 1.05 to 1.85) and with the 90-day window (OR = 1.49; 95% CI; 1.10 to 2.00); exposure to PM10 showed a protective effect in the 90-day window (OR = 0.69; 95% CI: 0.54 to 0.88). No association was found for the 60-day window, or in relation to NO_2_ exposure. In this step, the analysis was not adjusted for the variables of the previous levels.

**Table 3.**
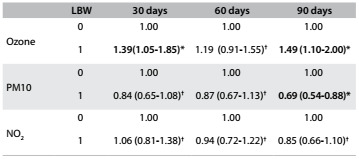
Odds ratios (OR) and 95% confidence intervals (95% CI) for presence (1) and absence (0) of low birth weight (LBW), according to concentrations of the pollutants ozone, particulate matter (PM10) and nitrogen dioxide (NO_2_), during the 30, 60 and 90-day windows prior to the newborns’ delivery, without adjustment and without distinction between the sexes. São José do Rio Preto Brazil - 2012-2013

*P < 0.05; ^†^P > 0.10.

Next, the ozone and particulate matter concentrations were adjusted for maternal educational level, marital status and number of consultations, for both sexes and separately for males and for females. It could be seen that there was no significant association for males in any exposure window. For both sexes, there were significant associations between exposure to ozone and LBW in the 30-day window and 90-day window. Significant exposures for females occurred in the 30-day window and the 90-day window. Maternal exposure to PM10 had a paradoxical protective effect when the analysis did not consider gender distinction (Table 4).

**Table 4.**
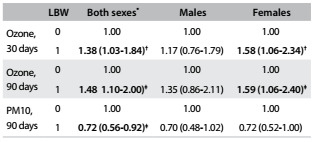
Odds ratios (OR) with 95% confidence intervals (95% CI) for presence (1) and absence (0) of low birth weight (LBW), according to ozone and PM10 exposure, singly, adjusted for marital status, maternal schooling and number of consultations during the 30 and 90-day windows, for both sexes and separately for males and females. São José do Rio Preto (SP), Brazil, 2012-2013

^*^Without distinction between the sexes; ^†^P = 0.03; ^‡^P = 0.02.

In the 30-day windows, without distinction between the sexes, maternal exposure to ozone increased the risk of low birth weight from 6.6% to 8.8%; regarding females, the risk increased from 8.2% to 12.3%.

Table 5 shows the odds ratios obtained from multipollutant analysis on maternal exposure and LBW. For both sexes and for female newborns, exposure to PM10 had a paradoxical protective effect; on the other hand, maternal exposure to ozone had a significant correlation for female newborns in 90 days window and for both sexes. In this window, maternal exposure to ozone increased the risk of low birth weight from 7.7% to 11.0% without distinction between the sexes; for females, the risk increased from 10.0% to 15.1%.

**Table 5.**
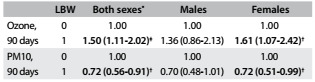
Odds ratios (OR) with 95% confidence intervals (95% CI) for presence (1) and absence (0) of low birth weight (LBW), according to particulate matter (PM10) and ozone exposure, adjusted for marital status, maternal schooling and number of consultations during the 90-day windows, for both sexes and separately for males and females. São José do Rio Preto (SP), Brazil, 2012-2013

^*^Without distinction between the sexes; ^†^P = 0.03; ^‡^P = 0.02.

## DISCUSSION

This was, to the best of our knowledge, the first Brazilian study on the effect of maternal exposure to air pollution during pregnancy and low birth weight, categorized according to the newborns’ sex. Maternal exposure to ozone in the 30 and 90-day windows prior to birth was seen to be associated with low birth weight, and this association was only with female newborns.

The prevalence of low birth weight in this study was 3.4%, which was lower than the prevalences found for Brazil (8.3%) and the state of São Paulo (9.2%) over this period. However, it is necessary to consider the exclusion factors that must have contributed to these results.^16^

The newborns with low birth weight in this study were significantly associated with lower and higher maternal ages^20^ (≤ 19 years and > 34 years), mothers living without a partner (single or separated), lower education level, fewer than seven prenatal visits and female sex. These findings coincide with those found by Haidar et al.^19^

The daily means for PM10, NO_2_ and ozone exposures in the 30, 60 and 90-day windows before the newborns’ delivery did not exceed the limits deemed to be acceptable through a state decree that established new air quality standards for the state of São Paulo, based on the guidelines established by WHO.^18^ These values for ozone were lower than those found in São José dos Campos (67.8 μg/m^3^),^6^ but higher than those found in Rio de Janeiro (44.5 μg/m^3^).^20^ For PM10, the values in São José do Rio Preto were higher than in São José dos Campos (35.2 μg/m^3^),^6^ and lower than in Rio de Janeiro (59 μg/m^3^).^20^

Bobak^7^ found an association with exposure to SO_2_ when this occurred in the third trimester of pregnancy, with OR = 1.27 for an increase of 50 μg/m^3^ when concentrations were of the order of 32 μg/m^3^ at the study site. Likewise, exposure to total suspended particles (TSP), which are particles of liquid or solid material suspended in the air in the shape of dust, mist or aerosol of less than 50 microns in diameter (which includes PM10), was significant during the first trimester of pregnancy, with OR = 1.18 for an increase of 50 μg/m^3^ in the concentration of this pollutant, for which the mean value was 72 μg/m^3^. These pollutants had not been quantified in São José do Rio Preto. PM10 showed paradoxical protective behavior in relation to exposure over the 90-day window before the newborns’ delivery, for both sexes and for males, with OR = 0.50 (95% CI: 0.29 to 0.86) and OR = 0.40 (95% CI: 0.16 to 0.98), respectively.

Medeiros and Gouveia^3^ identified an association between exposure to CO, PM10 and NO_2_ in the first trimester of pregnancy and LBW. In our study, it was not possible to find this association, possibly because the PM10 and NO_2_ concentrations were lower than those found in São Paulo, at the time of our study. In Rio de Janeiro too, no associations were identified between exposure to PM10, CO and NO_2_ and LBW, even with PM10 and NO_2_ concentrations higher than those found in São José do Rio Preto.^20^

In São José dos Campos (SP), a city in southeastern Brazil, maternal exposure to SO_2_ and ozone, over the 90 days before the newborns’ delivery was associated with LBW. The average concentrations were of the order of 6 μg/m^3^ for SO_2_ and 66 μg/m^3^ for ozone, and these mean concentrations of ozone were similar to those found in São José do Rio Preto.^6^ In a review, Maisonet et al. cited that exposure to SO_2_ was associated with LBW, but this pollutant is not monitored in São José do Rio Preto. On the other hand, NO_2_ levels were not significantly associated with LBW either in the study by Maisonet et al. or in São José do Rio Preto.^21^ In another review, Sram et al.^9^ reported that exposure to SO_2_ was significant, including exposure in the last trimester of pregnancy and that exposure to NO_2_ was only sometimes associated with LBW. Exposure to ozone was not associated with LBW or with intrauterine growth restriction in that review.

Liu et al. also did not find that exposure to ozone and NO_2_ was associated with LBW, either in the first or in the last month of pregnancy, after adjustment for maternal age, parity, newborn sex, gestational age and birth season, in a study carried out in Vancouver, Canada.^22^ The mean concentrations of these pollutants were of the order of 60 and 70 μg/m^3^ for ozone and NO_2_ respectively, and these values were not very different from those found in São José do Rio Preto.

On the other hand, a study carried out in southern California, a region with great variability of concentrations of air pollutants, showed that exposure to ozone had a significant deleterious role in relation to birth weight when this exposure occurred during the last trimester of pregnancy. Analysis on the pollutants individually showed a reduction of 37 g in birthweight secondary to the exposure to 70 μg/m^3^. When associated with PM10, an increase in ozone concentration of the order of 35 μg/m^3^ implied a significant reduction of 32 g in the newborns’ weight. This increase of 35 μg/m^3^ was associated with a decrease of 37 g in birth weight when the pollutants CO and NO_2_ were included in the model. Exposure to PM10 and NO_2_ in the third trimester was not associated with decreased weight among the infants.^4^

In a study conducted in Nova Scotia, Canada, where the average concentrations of ozone and PM10 were 45 μg/m^3^ and 17 μg/m^3^ respectively, it was not possible to identify any significant association between exposure to these pollutants in the third trimester of pregnancy and low birth weight. Exposure to PM10 in the first trimester of pregnancy was associated with low birth weight. The authors proposed a hypothesis of abnormal placental development and increased blood viscosity, due to an inflammatory response or the action of polycyclic aromatic hydrocarbons.^23^ Compared with our findings, the average concentrations found in Canada were lower than those found in São José do Rio Preto.

In the present study, without adjustment for other variables and without stratifying according to sex, ozone exposure was significant when it occurred in the 30 and 90-day windows before the newborns’ delivery. The chance of LBW was more evident with concentrations greater than 53 μg/m^3^. Exposure to ozone was shown to be a significant risk factor in the study by Salam et al.,^4^ with OR = 1.41, and in São José dos Campos (OR = 1.26).^6^ However, there was no association in other studies.^8^^,^^19^ In São José do Rio Preto, an association with ozone exposure was identified only among females.

We also observed that maternal exposure to PM10 showed paradoxical behavior over the 90-day period before birth, in that this exposure was reflected as a protective factor, with OR = 0.72 for both sexes and also for females. These values diverge from those found in other studies,^3^^,^^4^^,^^6^ even with similar concentrations. Our study considered homogeneous concentrations quantified at fixed monitoring stations. However, the distance from homes to roads with heavy traffic, distance-weighted traffic density (DWTD) and land use regression levels of particulate matter (LUR-PM10) were not considered as in the study by Habermann and Gouveia.^24^ These authors found that LBW was associated with DWTD and LUR-PM10 in the highest quartiles of exposure, with a significant linear trend of decrease in risk. We found that high maternal socioeconomic status, as assessed through higher educational level, had a protective effect against low birth weight. On the other hand, living in areas of higher vehicular traffic might not in fact give rise to greater expose to air pollution and the protection against LBW arising from better socioeconomic status might also be stronger than the effect of exposure to air pollution. This exposure may not have been sufficient to increase the risk of LBW for these mothers. Regarding particulate matter constituents, Basu et al.^25^ found that the largest reductions in birth weight were associated with exposure to vanadium, sulfur, sulfate, iron, elemental carbon, titanium, manganese, bromine, ammonium, zinc and copper, which were present as particulate matter constituents and were associated with increased risk of term LBW. In the stratified analysis according to sex, there were no differences.

No association between exposure to NO_2_ and LBW was found in our study. Our findings are consistent with those of some other studies,^1^^,^^2^^,^^3^ but are the opposite of those reported by Bell et al., who found that there was a decrease in weight of 9 g for each 10 μg/m^3^ increase in NO_2_ concentration.^15^

A significant association (P < 0.05) between exposure to ozone and LBW was identified in both the 30 and the 90-day windows before the newborns’ delivery. This analysis on ozone was adjusted for the variables of the previous levels (maternal education, marital status and prenatal number of consultations), considering both sexes and females separately. With inclusion of PM10 concentrations, exposure to ozone during the 90-day window was significant, considering both sexes and females separately.

Exposure to PM10 showed paradoxical protective behavior for both sexes, over the 30-day and 90-day windows before the newborns’ delivery. Salam et al. found that there was no significant association regarding exposure to PM10 during the last trimester of pregnancy. The mean concentrations found by these authors were similar to those in São José Rio Preto. The behavior of exposure to PM10 may have been an adjustment outcome for this pollutant when analyzed with ozone, or it may have been due to the composition and amount of material adsorbed on the particle, which depends on the study site.^4^

In the analysis according to the newborns’ sex, there was no significant association between maternal exposure to ozone and PM10 and low birth weight among males in any of the windows (30, 60 and 90 days) before delivery. For PM10, there was paradoxical behavior regarding exposure over the 30 and 90-day windows before delivery. For females, an association between maternal exposure and LBW was evident over the 30 and 90-day windows. The effect was larger over the 90-day window, in the second quartile (OR = 2.97). An association between ozone exposure and LBW had already been observed in São José dos Campos, Brazil,^6^ but not in other Brazilian studies. Regarding the effect among females, few studies have analyzed ozone and LBW and these either did not discriminate according to sex or the results were inconclusive.^15^^,^^26^ In a study conducted in Poland, a larger LBW effect was found among male newborns, but the pollutant analyzed was PM2.5.^13^ An association with exposure to ozone in the third trimester was identified by Ha et al. in Seoul (OR = 1.09; 95% CI: 1.04 to 1.14) when analyzed separately, but without any statistical significance in relation to other pollutants.^27^

The mechanisms that can lead to LBW because of ozone exposure are still unclear. In animals, particularly in pregnant rats, this effect can be modulated by inflammatory mediators, which make these animals more susceptible to acute lung inflammation because of impairment of the respiratory epithelial lining fluid. Since pregnant women have higher alveolar ventilation than non-pregnant women, the exposure level during pregnancy is likely to be higher, and similar inflammatory responses could be more pronounced in pregnant women. Moreover, inflammation due to ozone can result in release of products such as those associated with lipid peroxidation and circulating inflammatory cytokines. This may impair circulation and placental function and affect fetal growth.^4^ In healthy human volunteers exposed to ozone, effects were observed on biological markers, such as increased peripheral neutrophil levels and decreased ascorbic acid levels, upon exposure to 200 ppb (approximately 400 μg/m^3^) of ozone for two hours. In an extensive review, Ghosh et al. stated that there was strong evidence that females were at higher risk of LBW with adjusted odds ratios ranging from 1.07 to 1.62. In addition, there was some evidence to suggest that the effects of air pollution on LBW may be differential according to sex. However, they did not cite any possible mechanisms.^12^

Our study may have some limitations, such as: the concentrations of pollutants were taken to be homogeneous throughout the city and it was assumed that pregnant women were exposed to these concentrations in a similar way; no information was obtained on diseases during pregnancy that might have affect the newborns’ weight, such as smoking during this pregnancy, because the data source (SINASC), does not include this information; and, in addition, there was no information about the weight gain of the pregnant women. A further limitation may have arisen from the pregnant women’s address information, because it is possible that the mother’s residential address information and the length of time at this address (which would represent the length of time for which the mother was exposed to the pollutant concentrations studied) may not have corresponded to the reality. This study did not establish the cause between the exposure and outcome, but pointed towards possible associations.

Despite these limitations, the strength of this study is that, while pointing out the risks of exposure to air pollutants in the genesis of low birth weight, it identifies female newborns as more susceptible to impairment of birth weight, because of maternal exposure to ozone. This analysis suggests that in studies on the exposure of pregnant women to air pollutants, from which the outcome is low birth weight, it is necessary to separate the analysis between male and female newborns. Measures to reduce the concentration of pollutants in the air, particularly ozone, may reduce the prevalence of infants with low birth weight, thus reducing the risk of neonatal and infant mortality.

## CONCLUSION

Female newborns showed greater susceptibility to maternal exposure to air pollutants. Studies on low birth weight in relation to maternal exposure to air pollutants should deal with males and females separately.
